# Zinc provides neuroprotection by regulating NLRP3 inflammasome through autophagy and ubiquitination in a spinal contusion injury model

**DOI:** 10.1111/cns.13460

**Published:** 2020-10-09

**Authors:** Jia‐quan Lin, He Tian, Xiao‐guang Zhao, Sen Lin, Dao‐yong Li, Yuan‐ye Liu, Chang Xu, Xi‐fan Mei

**Affiliations:** ^1^ Department of Orthopedics The First Affiliated Hospital of Jinzhou Medical University Jinzhou China; ^2^ Department of Histology and Embryology Jinzhou Medical University Jinzhou China; ^3^ Department of Emergency The First Affifiliated Hospital of Jinzhou Medical University Jinzhou China

**Keywords:** autophagy, NLRP3 inflammasome, spinal cord injury, ubiquitination, zinc

## Abstract

**Aim:**

Spinal cord injury (SCI) is a serious disabling injury worldwide, and the excessive inflammatory response it causes plays an important role in secondary injury. Regulating the inflammatory response can be a potential therapeutic strategy for improving the prognosis of SCI. Zinc has been demonstrated to have a neuroprotective effect in experimental spinal cord injury models. In this study, we aimed to explore the neuroprotective effect of zinc through the suppression of the NLRP3 inflammasome.

**Method:**

Allen's method was used to establish an SCI model in C57BL/6J mice. The Basso Mouse Scale (BMS), Nissl staining were employed to confirm the protective effect of zinc on neuronal survival and functional recovery in vivo. Western blotting (WB), immunofluorescence (IF), and enzyme‐linked immunosorbent assay (ELISA) were used to detect the expression levels of NLRP3 inflammasome and autophagy‐related proteins. Transmission electron microscopy (TEM) was used to confirm the occurrence of zinc‐induced autophagy. In vitro, lipopolysaccharide (LPS) and ATP polarized BV2 cells to a proinflammatory phenotype. 3‐Methyladenine (3‐MA) and bafilomycin A1 (BafA1) were chosen to explore the relationship between the NLRP3 inflammasome and autophagy. A coimmunoprecipitation assay was used to detect the ubiquitination of the NLRP3 protein.

**Results:**

Our data showed that zinc significantly promoted motor function recovery after SCI. In vivo, zinc treatment inhibited the protein expression level of NLRP3 while increasing the level of autophagy. These effects were fully validated by the polarization of BV2 cells to a proinflammatory phenotype. The results showed that when 3‐MA and BafA1 were applied, the promotion of autophagy by zinc was blocked and that the inhibitory effect of zinc on NLRP3 was reversed. Furthermore, co‐IP confirmed that the promotion of autophagy by zinc also activated the protein expression of ubiquitin and suppressed high levels of NLRP3.

**Conclusion:**

Zinc provides neuroprotection by regulating NLRP3 inflammasome through autophagy and ubiquitination after SCI.

## Graphical abstract

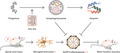

## 1 INTRODUCTION

Spinal cord injury (SCI) is a traumatic disease that is common worldwide and can lead to a reduction in the quality of life of patients. Unfortunately, there are no effective clinical treatments so far.^1^ SCI initiates a series of cellular and molecular events that include the inflammatory response.^2^ Posttraumatic inflammatory reactions have been reported to lead to progressive tissue damage in the secondary phase of SCI.^3^ After SCI, inflammation is triggered and induces the release a wide variety of cytokines, chemokines, and other inflammatory mediators which can reduce nerve cell survival.^4^ Martini first described inflammatory corpuscles in 2002 and found that they are related to a variety of autoinflammatory diseases.^5^ Secondary pathological stimulation can activate cellular receptors, which leads to the assembly of inflammatory corpuscles, the activation and mediation of the inflammatory response after injury, and the further aggravation of nerve tissue injury.^6^

NOD‐like receptor (NLR) family proteins are essential parts of the inflammatory response in secondary injury and 23 of these proteins have been found in humans so far.^7^ In particular, NLRP3 is the most widely studied member of this family at present.^8^ In the CNS, it is distributed in microglia and recent studies have also confirmed that it is obviously expressed and activated after spinal cord injury.^9^, ^10^ The NLRP3 inflammasome is a cellular polyprotein complex that serves as a platform for the activation of Caspase‐1 and the maturation of IL‐1β, a proinflammatory cytokine.^11^ Many regulatory mechanisms that attenuate inflammatory NLRP3 signals in multiple steps have been identified.^12^ Among these mechanisms, autophagy is induced by harmful substrates or the inactivation or selective removal of secondary signals. It can regulate the activation of the NLRP3 inflammasome.^13^ Although increasing evidence has shown that NLRP3 and the autophagy pathway are related through mutual regulation, the associated regulatory mechanisms have not been fully elucidated in spinal cord injury.

Recently, several studies have indicated that the NLRP3 inflammasome can be posttranscriptionally modified.^11^ Among posttranscriptional modifications, ubiquitination and autophagy represent new potential strategies for regulating NLRP3.^14^, ^15^ The ubiquitin proteasome system (UPS) and autophagy serve as two major clearance systems in humans, and their roles are removing necrotic tissue and abnormally accumulated proteins; the overactivated inflammasome is a potential target of these processes.^12^ Chong's result has shown that the ubiquitination of NLRP3 is associated with the activation of the NLRP3 inflammasome.^12^ The inhibition of NLRP3's activation led to a low level of ubiquitination.^12^ Autophagy is a basic catabolic reaction.^16^ In the beginning, vesicles with double membrane structure constitute primitive autophagic vesicles. Subsequently, the outer membrane fuses with the lysosome to form a functionally degrading autophagic vesicle which is commonly known as autophagy lysosome.^17^ The enzyme in the lysosome degrades the contents of the autophagic vesicle and the inner membrane to perform autophagy effect. Autophagy participates in many physiological activities in organisms, including the inhibition of inflammatory chemokine release.^18^ Therefore, a better understanding of the mutual regulatory interactions between autophagy and the NLRP3 inflammasome is essential for discovering new therapeutics for spinal cord injury.

Zinc gluconate is an organic complex containing zinc that is formed by the complexation of glucose and zinc.^19^ When ingested by bodies, zinc gluconate can be completely dissolved in the blood and cause the release of zinc, which is due to its physical and chemical properties. Zinc plays an important role in many physiological and pathological events, such as immunity, proliferation, and apoptosis.^20^ Many studies have shown that zinc has antioxidative effects.^21^ Interestingly, researchers found that zinc shows the potential to increase autophagy.^22^ The activation of autophagy promotes the formation of a microenvironment by reducing cell apoptosis and restores cell survival by promoting the phagocytosis of dead and inactivated cells and the excessive degradation of abnormal proteins in cells.^23^ Welling's research showed that zinc deficiency compromises the function of primarily T cells as well as that of several other immune cells, such as microglia in the CNS,^24^ and their follow‐up studies also suggested that zinc inhibits IL‐1β in a dose‐dependent manner, indicating that zinc has a direct anti‐inflammatory effect.^25^ Furthermore, our previous studies have found that zinc promotes SCI recovery not only by elevating brain‐derived neurotrophic factor (BDNF) levels but also by promoting granulocyte colony stimulating factor (G‐CSF), which can protect neurons from death caused by secondary injuries.^26^, ^27^, ^28^ Although the neuroprotective effect of zinc has been confirmed, the anti‐inflammatory effect of zinc in acute SCI has not been investigated.

In the present study, we demonstrated that zinc significantly protects mice against SCI by inhibiting NLRP3 inflammasome expression. Further studies have shown that zinc induces autophagy and targets NLRP3 for degradation by ubiquitination, thereby suppressing the NLRP3 inflammasome. In general, these findings could help to guide clinical decisions regarding the use of zinc as a treatment and improve patient quality of life.

## 2 MATERIALS AND METHODS

### 2.1 Animals

The experiments were performed using adult C57BL/6J mice (half males and half females, 20‐25 g, 8 weeks old) in accordance with the Guide for the Care and Use of Laboratory Animals (National Research Council, 1996) and the Guidelines and Policies for Rodent Survival Surgery established by the Animal Care and Use Committee of Jinzhou Medical College and were approved by the Animal Care and Use Committees of Jinzhou Medical College. These animals were maintained at 22°C‐24°C with a 12‐h/12‐h light‐dark cycle with free access to food and water for 1 week to allow acclimatization to the environment prior to experimentation.

### 2.2 SCI model and administration of zinc

All animals were randomly divided into three groups: the sham group, SCI + vehicle group, and SCI + zinc group. We chose to use the method described by Allen to establish a spinal contusion injury model.^29^ We anesthetized all animals with 10% chloral hydrate (0.3 mL/100 g) combined with 0.03 mg/kg fentanyl,^30^ and a laminectomy was performed at the T9‐T10 level without damaging the dura mater. Moderate contusive injury was made by dropping an impactor (RWD, Cat#Model Ⅲ, USA, 2 mm diameter, 12.5 g, 20 mm in height) onto the surface of the T9‐T10 spinal cord.^26^ The muscles, fascia, and skin were sutured layer by layer and disinfected with iodine, and the mice were placed at a constant suitable temperature in a moderately illuminated chamber. Sham group mice underwent the same surgical procedure except contusion. Postoperative monitoring included manual bladder emptying three times daily until reflexive bladder control was reestablished. Two hours after the injury, zinc gluconate was first injected into the zinc group (30 mg/kg i.p; Biotopped, Beijing, China), and 30 mg/kg zinc (0.1 mL) (Figure 2A) was then given daily until day three; the vehicle group mice were injected with an equivalent volume of isotonic glucose (0.1 mL) as a control.^26^

### 2.3 Western blot analysis

Tissue (a 1.0 cm piece from the injury epicenter of the spinal cord) or cells were physically sheared on ice using RIPA lysis containing PMSF buffer for 30 minutes to obtain protein. The debris was removed by centrifugation at 12 000 rpm (25 minutes, 4°C), and the supernatant was immediately stored at −80°C. Then, the proteins obtained from the tissues or cells were quantified by BCA reagents, and equivalent amounts of protein (40 µg) were separated on 8%‐15% SDS‐PAGE gels, transferred to polyvinylidene fluoride (PVDF) membranes and incubated with the appropriate primary antibodies overnight. The PVDF membranes were washed with TBST and incubated with secondary antibodies (1:5000; Proteintech, USA) for 90 minutes at room temperature. Finally, the bands were detected by BeyoECL Plus, and the signals were visualized via a Imaging System. Primary antibodies were as follows: anti‐LC3B (1:1000; Abcam, UK); anti‐p62 (1:1,000; Abcam, UK); anti‐Beclin1 (1:1000; CST, USA); anti‐NLRP3 (1:1000; Affinity, USA); anti‐ASC (1:100; Affitiny, USA); anti‐Caspase‐1 (1:100; CST, USA), anti‐IL1β (1:1,000; Abcam, UK); anti‐cl‐IL1β (1:1,000; Abcam, UK); anti‐IL6 (1:1,000; Abcam, UK); anti‐Ubiquitin (1:1000; Abcam, UK); anti‐β‐Actin (1:1000; Proteintech, USA); anti‐GAPDH (1:1000; Proteintech, USA).

### 2.4 Enzyme‐linked immunosorbent assay

The levels of IL1, IL‐6, and NLRP3 were detected by following the protocol of a commercial ELISA kit. Briefly, the sample, the standard, and a HRP‐labeled antibody were added to microwells coated with the antibody, incubated at 37°C and washed thoroughly. The color was developed with TMB substrate, which was converted to blue by peroxidase catalysis and ultimately converted to yellow by the action of acid. The intensity of the color was positively correlated with the expression of the target protein in the sample. The absorbance (OD value) was measured at 450 nm with a microplate reader, and the target protein content in the final sample was calculated from the standard curve.

### 2.5 Tissue preparation

Animals were anesthetized as described above at a specific time after operation and then intracardially perfused with 0.9% normal saline followed by 4% sodium methyl benzoate. Then, 0.5 cm‐long pieces of tissue from above and below the epicenter of the damaged spinal cord were collected. The collected spinal cord tissues were fixed for 3 days and then moved to 30% sucrose in 4% paraformaldehyde until they sunk. Frozen sections (10 µm thick) of the region of the spinal cord that was 3 mm away from the injury epicenter were prepared with a cryostat and stored at −80°C.

Paraffin section's preparation is similar to frozen section. After fixed in 4% paraformaldehyde, the tissues were collected from the same position and length and put it into the dehydration box. Next, sequentially gradient alcohol in the dehydrator for dehydration: 75% alcohol 4 hours, 85% alcohol 2 hours, 95% alcohol 1 hour, anhydrous ethanol twice 30 minutes each time, alcohol benzene 5‐10 minutes, xylene twice 5‐10 minutes each time, finally dipped in wax for 3 hours. After the dehydration process is completed, embed the wax‐soaked tissue in the embedding machine. Finally, place the wax block on a paraffin microtome and got the slices (4 µm thick) and stored at −4°C.

### 2.6 Immunofluorescence analysis

Mice were sacrificed at the third day after zinc administration. The tissue was prepared as described above. The frozen sections were dried at room temperature for 2 hours. The sections were then incubated at room temperature with 5% normal goat serum containing 0.3% Triton X‐100 for 2 hours. Then, 1x PBS was used to wash the section three times for 5 minutes each. Primary antibody was added, and the sections were incubated with gentle shaking for 12 hours at 4°C. On the following day, 1x PBS was used to wash the section three times for 5 minutes each. Next, the sections were incubated with secondary antibody for 8 hours on a shaker at 4°C. 4'6‐Diamidino‐2‐phenylindole (DAPI) solution was used to stain the nuclei. The slices were selected from 0.5 cm‐long pieces of tissue from above and below the epicenter of the damaged spinal cord. For each mouse, select every other continuous section and count three sections in total. In the ventral horn area on both sides of the spinal cord, a 200 × 200 µm area was selected to count the number of neuron‐positive cell, IBA‐1‐positive cell, and NLRP3‐positive cell. The antibodies used are listed as followed: anti‐NeuN (1:1000; abcam, UK), anti‐Iba‐1 (1:500; Wako, JP), and anti‐NLRP3 (1:250, Affinity, USA).

### 2.7 Nissl staining

The tissue was prepared as described above. We follow the procedure of the Nissl staining kit (Solarbio, Cat#G1436, CN) for staining and the brief description is as follows: First, the paraffin sections were dried at room temperature for 2 hours. Next, the sections were dewaxed twice for 10 minutes each in xylene. Then, the sections were hydrated in 100% ethanol, 95% ethanol, and 70% ethanol for 5 minutes. The slices were rinsed in tap water and later in distilled water. Finally, the sections were stained with 0.5% cresol violet solution for 3‐10 minutes and immediately rinsed in distilled water. Next, the sections were differentiated in 95% ethanol for 20 minutes. Then, 100% ethanol was used to dehydrate the sections twice for 5 minutes each. Finally, the sections were washed in xylene for 10 minutes and permanently sealed, and the numbers of Nissl‐positive cells were observed and counted under a microscope. The slices were selected from 0.5‐cm‐long pieces of tissue from above and below the epicenter of the damaged spinal cord. For each mouse, select every other continuous section and count three sections in total. In the ventral horn area on both sides of the spinal cord, a 200 × 200 µm area was selected to count the number of classical Nissl bodies and the average was calculated. The classic Nissl‐positive motor neuron is plaque‐like as described in the previous article.^31^

### 2.8 Transmission electron microscope analysis

The mice were sacrificed on the third day after zinc administration and then subjected to special tissue processing required for TEM analysis. The brief description is as follows: All animals were anesthetized as described above at a specific time after operation. After fixing the mouse, infuse the animal with 50 mL of pre‐cooled 3% glutaraldehyde fixative solution through the left ventricle. A sharp blade was used to quickly and completely remove a piece of the damaged spinal cord and the tissue was placed in glutaraldehyde fixative for long‐term storage. Next, the tissue sample was removed from the fixative solution, washed three times with PBS for five minutes each, and then fixed in 1% acetic acid fixative for 1 hour. After washing three times with PBS, the tissues were placed in 50%, 70%, 80%, 90%, and 100% acetone and double distilled water for dehydration. After dehydration was completed, a permeabilization buffer with a 3:1 ratio of embedding agent to acetone was prepared, and the tissue was permeabilized for 2 hours. Next, embedding was performed under a temperature gradient in an oven at 45°C for 24 hours and at 60°C for 48 hours. After successful embedding, 60‐nm tissue slices were cut with an ultrathin microtome, stained with 1% uranium acetate‐lead citrate, and finally observed the autophagosome under a transmission electron microscope. A typical autophagosome is a vesicle structure with a double‐layer membrane around 500 nm. The slices were selected from 0.5 cm‐long pieces of tissue from above and below the epicenter of the damaged spinal cord. Observe the number of typical autophagosomes around a single cell nucleus under the EM4200X field of view. Three consecutive discontinuous slices were taken for each tissue sample, three fields of view were selected for statistics and the average was taken.

### 2.9 Behavioral assessment

On days 1, 3, 7, 14, 21, and 28, the Basso Mouse Scale (BMS) locomotor rating scale was used to evaluate the functional recovery of the injured animals. Scoring ranging from 0 to 9 points (9 meaning completely normal and 0 meaning completely paralyzed) were assigned. The mice were placed in an open field arena and allowed to move freely for 5 minutes. The range of motion, coordination, paw posture, trunk stability, and posture of the hind limbs and tail of the mice were observed and scored. Observations and scoring were performed independently by three examiners who were unaware of the grouping of the mice, and the experiment was repeated three times.

### 2.10 Cell culture and treatment

BV2 cells (a microglia cell line) were cultured in DMEM supplemented with 10% FBS and 50 µg/mL penicillin/streptomycin at 37°C and 5% CO_2_. BV2 cells were stimulated with LPS (1 µg/mL) and ATP (5 mmol/L) for 6 hours to induce a proinflammatory phenotype involving NLRP3 protein expression. After successfull induction of an increase in NLRP3 inflammasome expression, the cells were treated with zinc gluconate (100 µmol/L) for 6 hours. LPS‐primed BV2 cells were cultured with 3‐MA (5 mmol/L) or bafilomycin A1 (100 nmol/L) for 2 hours before being treated with zinc. LPS‐primed BV2 cells were treated with MG‐132 (10 µmol/L) 2 hours before collection. 3‐MA and Bafilomycin A1 are two autophagy inhibitors widely used in research. The former function is to prevent the formation of vesicle double‐layer membrane, while the latter is to prevent the binding of autophagosomes and lysosomes to inhibit autophagy.^16^ In the study of ubiquitination modification, MG‐132 is a class of highly effective inhibitors that can inhibit cell ubiquitination.^32^

### 2.11 Confocal fluorescence microscopy of cells

Cells were cultured in confocal culture dishes in the manner described above, removed, placed on a slide at 4°C, washed three times with 1x PBS for 5 minutes each, fixed with 4% PFA for 30 minutes, washed again with PBS three times, permeabilized with 1‰ Triton X‐100 for 30 minutes, blocked with sheep serum for 2 hours, washed with PBS three times, and incubated with an appropriate dilution of primary antibody in a 4°C refrigerator overnight. On the second day, after three washes in PBS, the cells were incubated with a suitable dilution of secondary antibody at room temperature for 4 hours, washed with PBS and incubated with DAPI for 30 minutes. Finally, the cells were incubated with ghost dye at room temperature in the dark, incubated for 30 minutes, and observed and imaged with a high‐resolution confocal microscope.

### 2.12 Immunoprecipitation assay

A total of 100 µL 1x IP lysis buffer (containing protease inhibitor) was added to the collected cell, and the cells were lysed on ice for 30 minutes. The supernatant was centrifuged at 12 000 *g* for 30 minutes. After that, the precleaned lysates were mixed with 1 µg primary antibody and 50 µL protein A/G beads. The mixture was shaken gently at 4°C and incubated overnight. After the immunoprecipitation reaction, the samples were centrifuged at a speed of 3000 *g* for 5 minutes at 4°C to collect the protein A/G beads at the bottom of each tube. The supernatant was carefully removed. The protein A/G beads were washed with 1 mL 0.1x IP buffer three to four times. A total of 50 µL of 2x SDS sample buffer was added, and the samples were boiled for 10 minutes. Finally, 20 µL of each sample was separated by SDS‐PAGE for Western blot analysis.

### 2.13 Statistical analysis

SPSS 21.0 was used for analysis, and all the data are expressed as the means ± SD Shapiro‐Wilk test was used to assess data distribution. Two experimental groups were analyzed by using Mann‐Whitney U test, when more than two groups were compared, one‐way analysis of variance (ANOVA) followed by Bonferroni post hoc test was used, except for BMS scoring analyzed by two‐way analysis of variance (ANOVA) following Tukey's post hoc test. The Kruskal‐Wallis test was used instead when the variance was not equal. In all analyses, *P* < .05 indicated a statistically significant difference.

## 3 RESULTS

### 3.1 NLRP3 inflammasome cytokine activation after SCI

**FIGURE 1:**
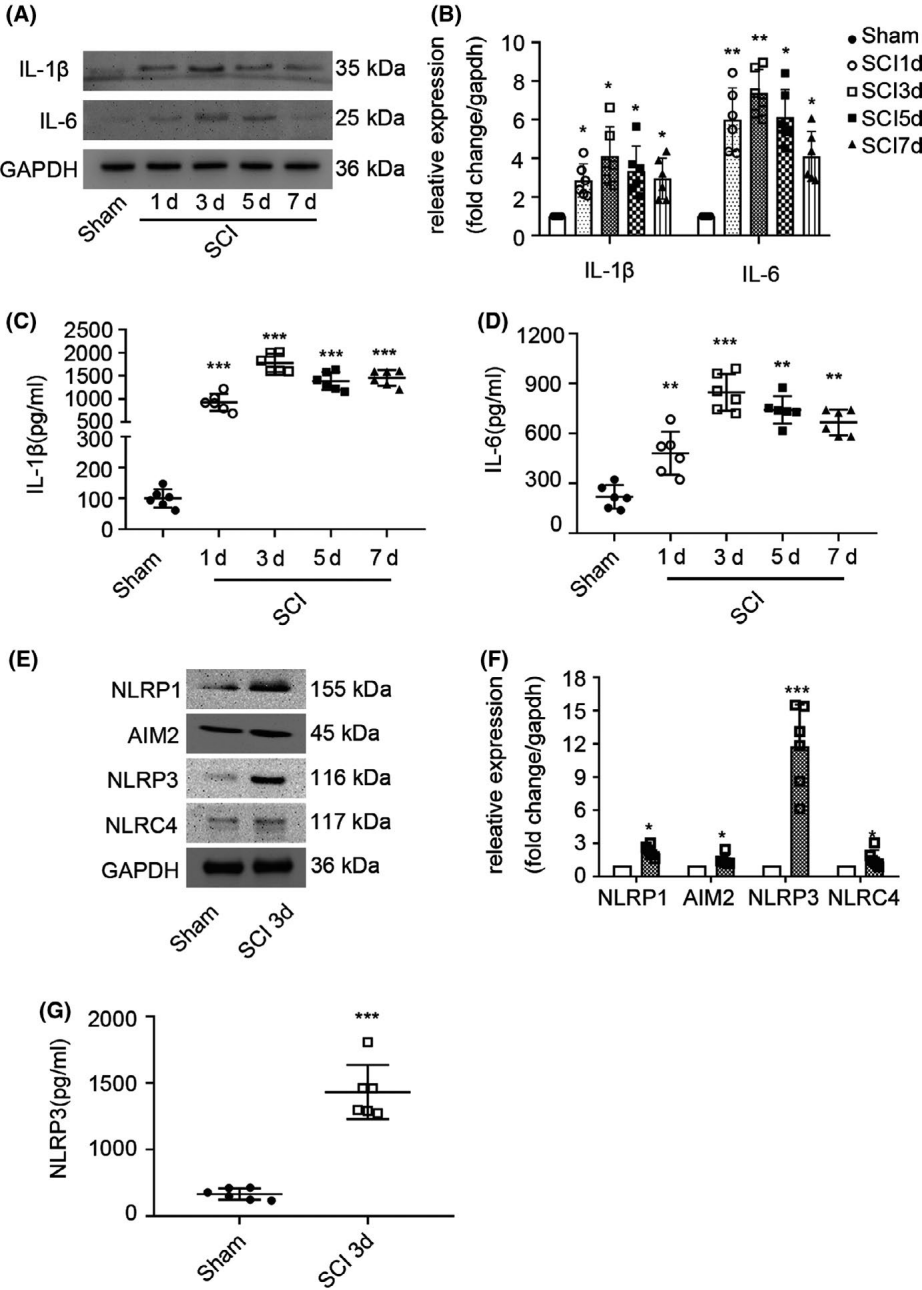
NLRP3 inflammasome cytokine activation after SCI (A) WB was used to detect the inflammatory cytokines. (B) The analysis of IL‐1β and IL‐6 after the operation of day 1, 3, 5, 7 of the acute spinal cord injury (n = 6, all the data are expressed as means ± SD and two‐way ANOVA followed by Turkey's post hoc test was applied). (C, D) The analysis of IL‐1β and IL‐6 examined by ELISA (n = 6, all the data are expressed as means ± SD and two‐way ANOVA followed by Turkey's post hoc test was applied). (E, F) The analysis of the expression of NLRP1, AIM2, NLRP3, and NLRC4. (G) NLRP3 was detected by ELISA at the day of three after operation (n = 6, all the data are expressed as means ± SD, Mann‐Whitney U test was applied). * Representative comparison with Sham group, *P < .05; **P < .01; ***P < .001

Inflammatory cytokines were detected in injured spinal cord segment samples by WB (Figure 1A) and ELISA on days 1, 3, 5, and 7 after trauma, and sham group samples were used as negative controls. The protein expression of IL‐1 and IL‐6 reached a peak on the third day after SCI (Figure 1B). ELISA analysis showed that the levels of IL‐1 and IL‐6 reached the highest point on the third day levels (Figure 1C,D). On this basis, we analyzed the expression of the four main inflammasomes, the NLRP1, AIM2, NLRP3, and NLRC4 inflammasome, in the spinal cord (Figure 1E) by WB, and the results showed that the NLRP3 inflammasome was the most prominently expressed inflammasome (Figure 1F). ELISA analysis showed that the level of NLRP3 in the SCI group was higher than that in the sham group on the third day (Figure 1G).

### 3.2 Zinc promotes the recovery of motor neurons following SCI

**FIGURE 2:**
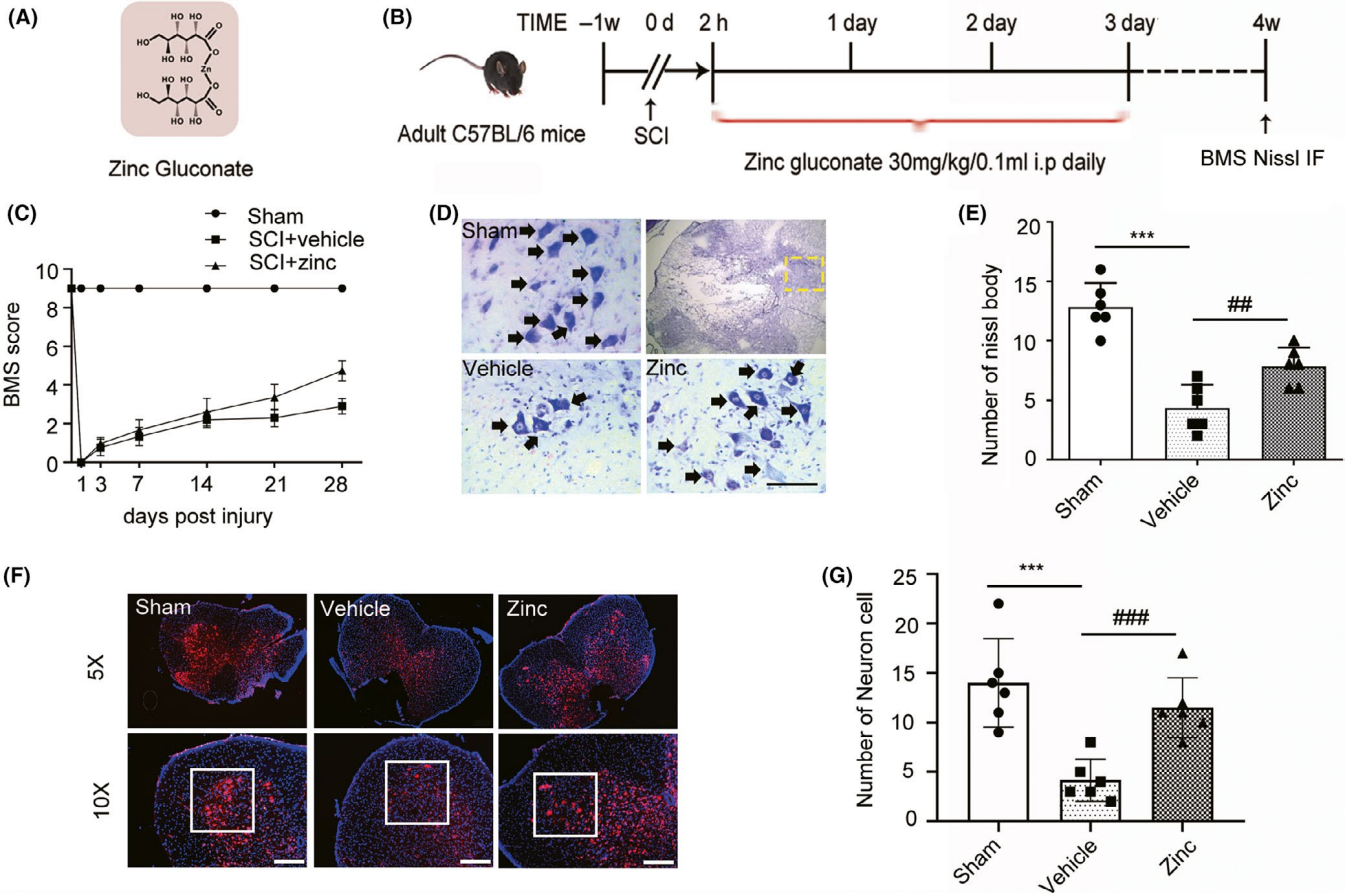
Zinc promotes the recovery of motor neurons following SCI. (A) Structural formula of zinc gluconate. (B) The design of the study. (C) BMS scores was performed to evaluate the motor function recovery of Sham, SCI + Vehicle, SCI + zinc group at the day of 1, 3, 5, 7, 14, 21, 28 (n = 20, all the data are expressed as means ± SD, two‐way ANOVA followed by Turkey's post hoc test was applied). (D, E) Nissl staining were used to observe the number of ventral motor neuron and counting the number of the Nissl‐positive neuron cells (n = 6, all the data are expressed as means ± SD one‐way ANOVA followed by Bonferroni post hoc test was used). The yellow rectangular box indicates where the count is observed (Scale bar = 100 µm). (F, G) IF was used to detect the number of neurons in the ventral horn of the spinal cord and the analysis of result in (f).(n = 6, all the data are expressed as means ± SD one‐way ANOVA followed by Bonferroni post hoc test was used, Scale bar = 100 µm). * represents comparison between SCI + vehicle group and sham group; # represents comparison between SCI + zinc group and SCI + vehicle group * or # means *P* < .05; **or ## means *P* < .01; *** means *P* < .001

Zinc gluconate is a kind of organic compound (Figure 2A). According to the design of this study (Figure 2B), we evaluated the neuroprotective effects of zinc by using BMS scores. The scores of the vehicle group were not statistically different from those of the zinc treatment group until three weeks postcontusion (Figure 2C). To confirm these results, Nissl staining and IF analysis were used to detect the living neuron cells (Figure 2D,F). The number of living neurons in the anterior horn of the zinc group was higher than that in the vehicle group (Figure 2E,G).

### 3.3 Zinc promotes the expression of autophagy‐related proteins after SCI

**FIGURE 3:**
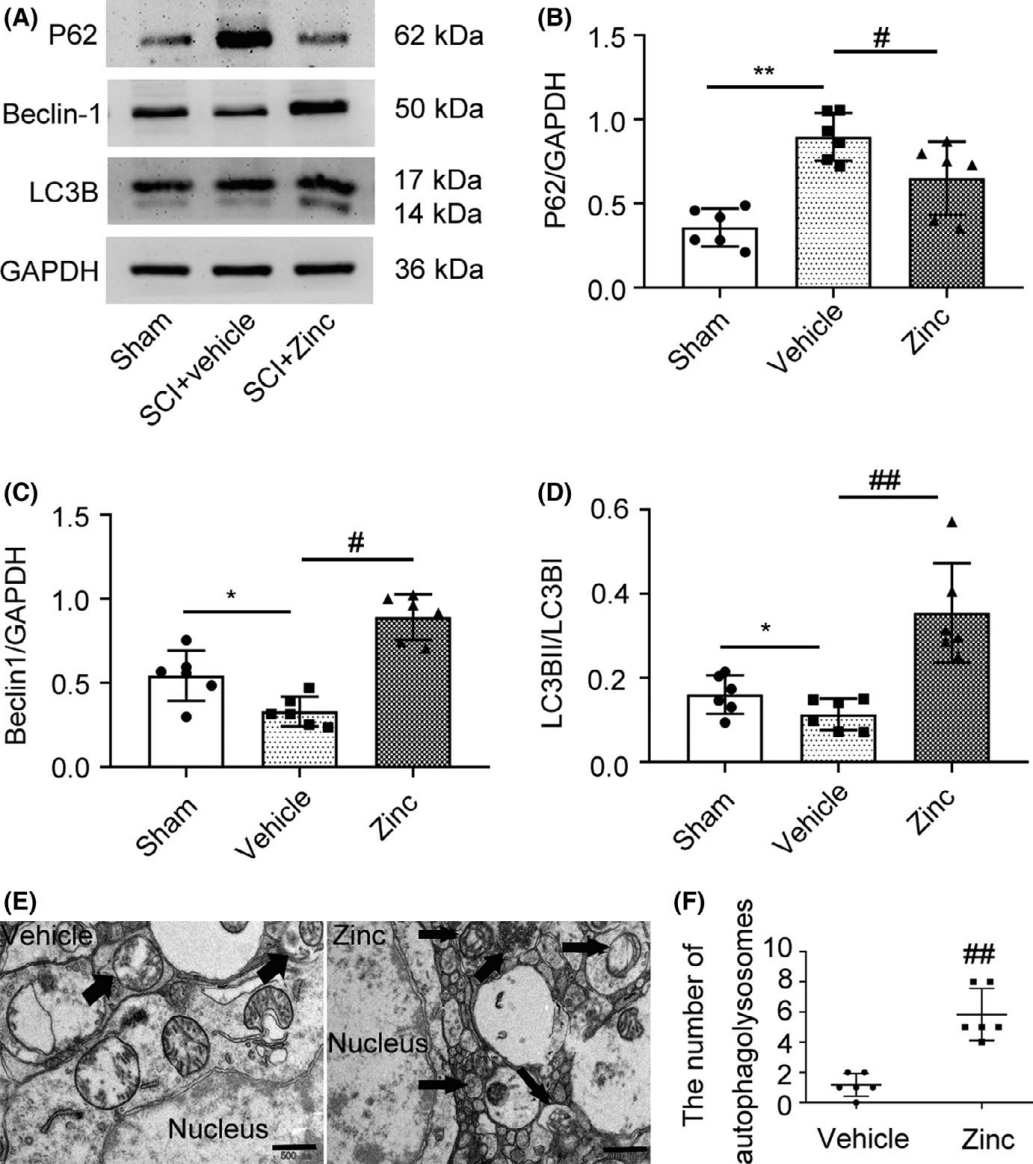
Zinc promotes the expression of autophagy‐related proteins after SCI Mice were sacrificed on the third day after zinc administration. (A) Western blot was used to detect the autophagy‐relative protein p62, Beclin1 and LC3B of sham, Sci + vehicle Sci + zinc group (n = 6, all the data are expressed as means ± SD one‐way ANOVA followed by Bonferroni post hoc test was used). (B‐D) The results of analysis about autophagy‐relative protein was showed. (E, F) Observe and count the number of autophagic lysosomes after zinc treatment by TEM (n = 3, Scale bar = 500 nm, all the data are expressed as means ± SD, Mann‐Whitney U test was applied). * represents comparison between SCI + vehicle group and sham group; # represents comparison between SCI + zinc group and SCI + vehicle group * or # means *P* < .05; ** or ## means *P* < .01

To investigate the potential mechanism of the neuroprotective effects of zinc, we examined the levels of the autophagy‐related proteins P62, LC3B, and Beclin1 by using Western blot analysis (Figure 3A). It is worth clarifying that the protein expression level of LC3BII/I and Beclin1 reflects the activity of autophagy. The level of P62, unlike LC3BII/LC3BI and Beclin1, is negatively correlated with autophagy. The results showed that P62 expression was suppressed in the zinc‐treated group compared to the vehicle group (Figure 3B). The vehicle group showed a decrease in autophagy compared to that the sham group, while zinc treatment caused a significant rise in the protein expression of LC3BII and Beclin1 compared to that in the vehicle group (Figure 3C,D), indicating that zinc has the potential ability to promote the autophagy after SCI. At the same time, we performed transmission electron microscopy on injured spinal cord tissues (Figure 3E), and the results showed that the number of autophagosomes increased significantly after zinc treatment (Figure 3F).

### 3.4 Treatment with zinc suppresses the NLRP3 inflammasome after SCI

**FIGURE 4:**
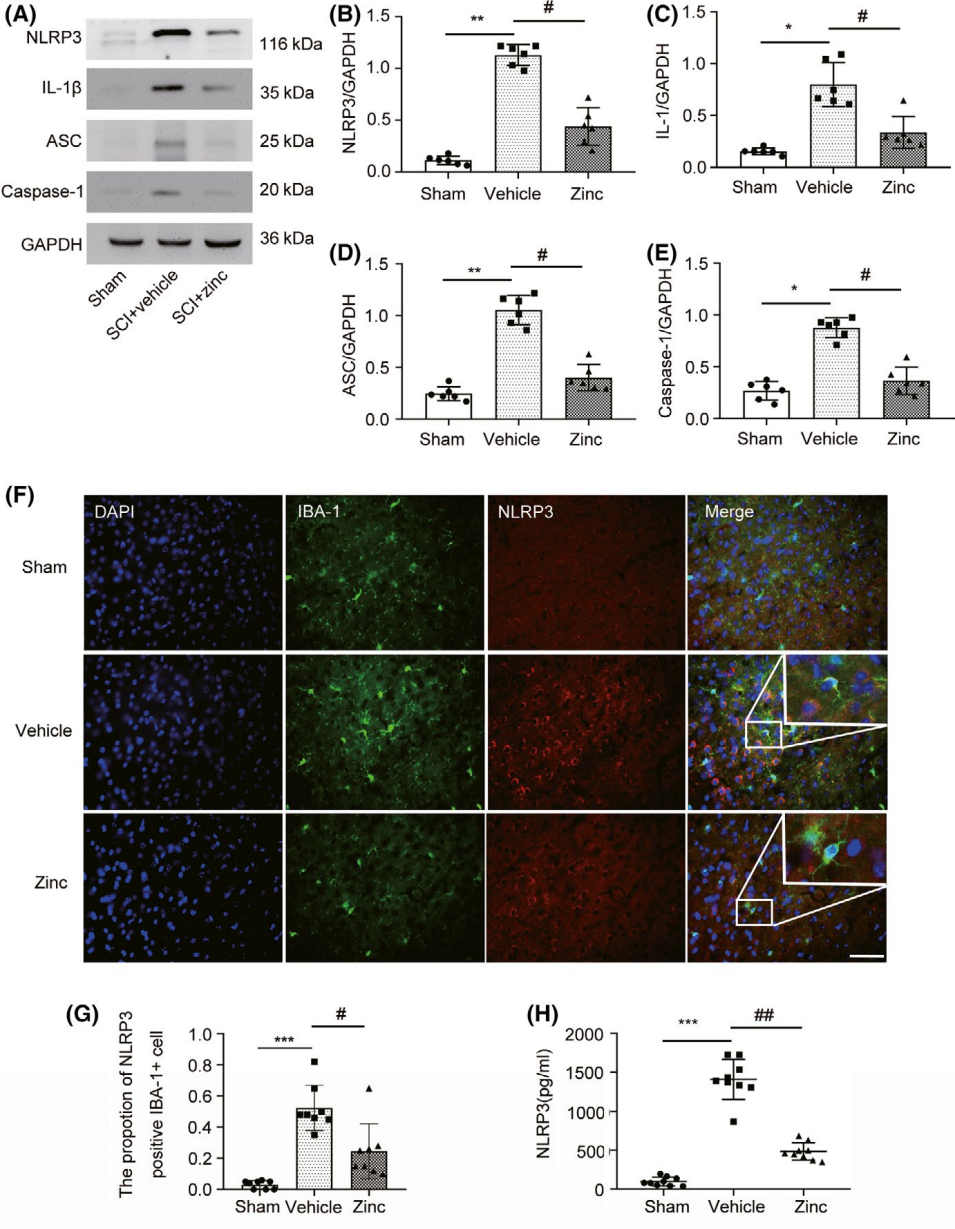
Treatment with zinc suppresses the NLRP3 inflammasome after SCI Mice were sacrificed on the third day after zinc administration.(A) Representative Western blot analysis of NLRP3 inflammasome in Sham, SCI + vehicle and SCI + zinc group (n = 6). (B‐E) The result of WB analysis of NLRP3, ASC, Caspase‐1, IL‐1β from each group. (F) Immunofluorescence staining was used to detect the level of NLRP3 from each group (n = 8, scale bar = 50 µm). (G) Statistical analysis of immunofluorescence staining for positive expression of NLRP3 in IBA‐1+ cells from each group. (H) NLRP3 was detected by ELISA among the Sham, SCI + vehicle, and SCI + zinc group (n = 8, all the data are expressed as means ± SD one‐way ANOVA followed by Bonferroni post hoc test was used). * represents comparison between SCI + vehicle group and sham group; # represents comparison between SCI + zinc group and SCI + vehicle group * or # means *P* < .05; ** or ## means *P* < .01.*** means *P* < .001

Further study showed that zinc treatment inhibited NLRP3 inflammasome activation in microglia. WB was used to detect the NLRP3 inflammasome‐related proteins NLRP3, ASC, IL‐1β, and Caspase‐1 (Figure 4A). Compared with sham group mice, mice with spinal cord injury expressed high levels of NLRP3, ASC, IL1, and Caspase‐1, and this increase in protein expression was suppressed by zinc treatment (Figure 4B‐E). IF analysis of NLRP3 was carried out to confirm these results (Figure 4F). The proportion of NLRP3‐positive IBA‐1 cell in the zinc group tended to be decreased compared to that in the SCI group (Figure 4G). ELISA for NLRP3 showed that zinc treatment significantly inhibited the expression of NLRP3 after SCI (Figure 4H).

### 3.5 Zinc inhibits the NLRP3 inflammasome in LPS‐treated BV2 cells by activating autophagy

**FIGURE 5:**
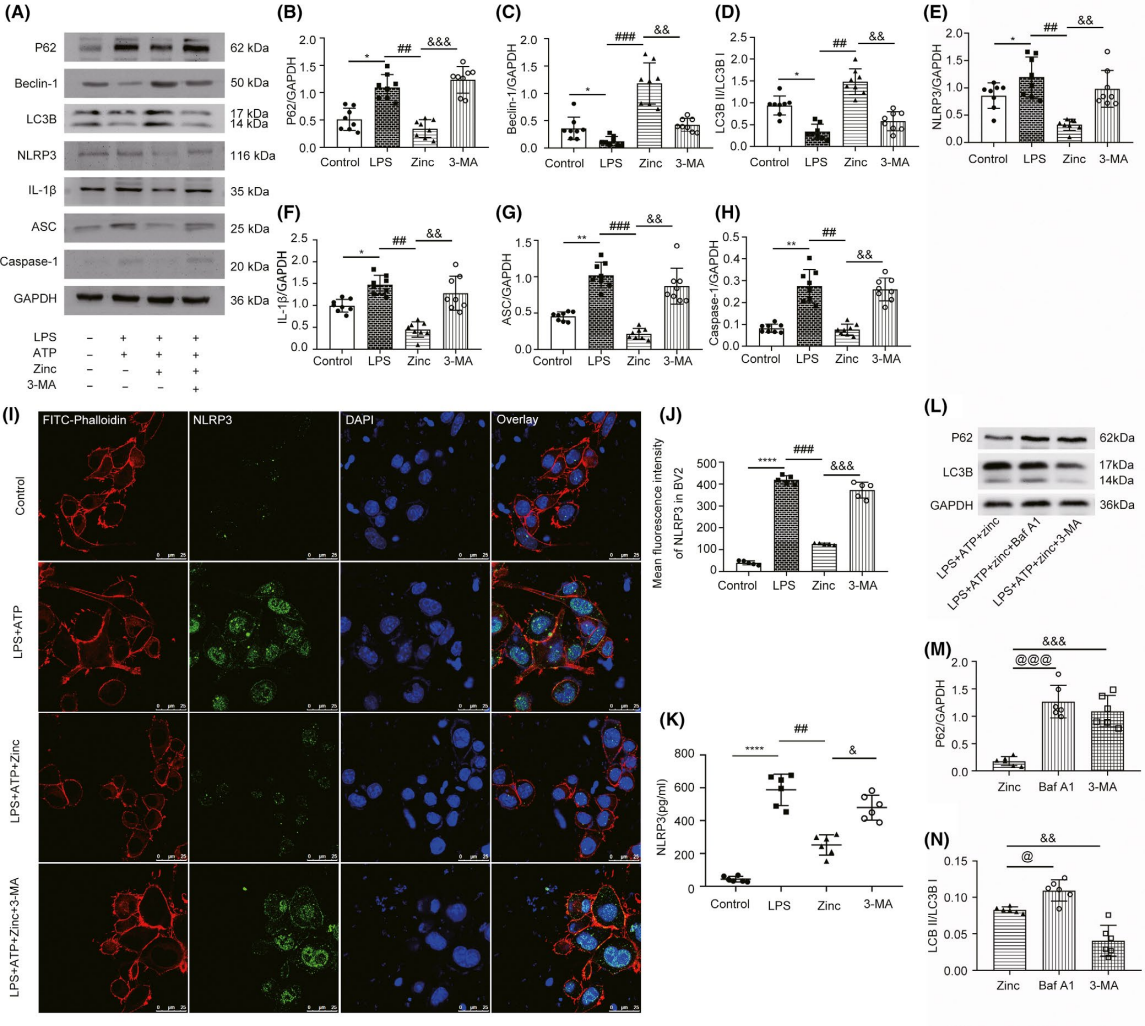
Zinc inhibits the NLRP3 inflammasome in LPS‐treated BV2 cells by activating autophagy (A) Representative Western blot analysis of NLRP3, IL‐1β, Caspase‐1, ASC and autophagy‐relative protein:P62, Beclin1, LC3B in control, LPS + ATP, LPS + ATP + zinc, LPS + ATP + zinc + 3‐MA group (n = 6, all the data are expressed as means ± SD one‐way ANOVA followed by Bonferroni post hoc test was used). (B‐H) The result of Western blot analysis. (I) Laser scanning confocal microscopy (LSCM) was used to detect the NLRP3's expression in BV2 cells (n = 8, scale bar = 25 µm, all the data are expressed as means ± SD one‐way ANOVA followed by Bonferroni post hoc test was used). (J) Statistical analysis of mean fluorescence intensity of NLRP3 in BV2 from each group. (K) NLRP3 was detected by ELISA among the control, LPS + ATP, LPS + ATP + zinc, LPS + ATP + zinc + 3‐MAgroup. (L‐N) Representative WB analysis of NLRP3 and LC3B after treatment of 3‐MA and BAF A1 (n = 6, all the data are expressed as means ± SD one‐way ANOVA followed by Bonferroni post hoc test was used). * represents comparison between LPS + ATP group and control group; # represents comparison between LPS + ATP + zinc group and LPS + ATP group; & represents comparison between LPS + ATP + zinc + 3‐MA group and LPS + ATP + zinc group; @ represents comparison between LPS + ATP + zinc + Baf A1 group and LPS + ATP + zinc group. *, #, & and @ means *P* < .05; **, ## and && means *P* < .01; ***, ###, &&& and @@@ means *P* < .001

To investigate the complex relationship between autophagy and NLRP3 after zinc treatment, we successfully induced an inflammatory phenotype involving the NLRP3 inflammasome in BV2 cells with LPS and ATP for 6 hours, as detected by WB and ELISA (Figure S1). The expression of NLRP3 and ASC did not change significantly when only LPS was applied, and NLRP3 was successfully induced after additional treated with ATP (Figure S1B‐D). The MTT assay was used to confirm the protective effect of zinc in LPS‐primed BV2 cells (Figure S2A,B). As mentioned earlier, treatment with excess zinc causes some cytotoxicity.^26^ When the concentration of zinc was greater than 120 µmol, the survival of BV2 cells was significantly inhibited (Figure S2A). In addition, we used JC‐1 staining to evaluate the mitochondrial membrane potential of BV2 cells under different treatment conditions (Figure S2C). The results showed that the zinc treatment group can significantly enhance the aggregation of JC‐1 and mitochondrial matrix, indicating that the administration of zinc significantly improves the mitochondrial function of the cells and reduces the level of apoptosis (Figure S2D). The levels of NLRP3 inflammasome‐ and autophagy‐related proteins were examined by WB (Figure 5A). 3‐MA is a highly effective autophagy inhibitor in BV2 cells. In the zinc‐treated group, the expression level of P62 was lower than that in the LPS and ATP‐treated group, and the effect of 3‐MA increased the level of P62 (Figure 5B). The same trend was also observed for the protein expression levels of NLRP3, ASC, IL‐1, and Caspase‐1. The effect of zinc also enhanced the expression of autophagy‐related proteins (Figure 5E‐H). The levels of LC3B and Beclin1 in the zinc‐treated group were significantly higher than those in the LPS and ATP‐treated group (Figure 5C,D). We also used cell confocal microscopy to observe the expression of NLRP3 in BV2 cells (Figure 5I). After zinc application, the average fluorescence intensity was significantly lower than that in the LPS‐treated BV2 cells. Additionally, 3‐MA significantly increased NLRP3 levels (Figure 5J). NLRP3 levels in the BV2 cell culture medium were detected by ELISA to confirm the above changes. As expected, the level of NLRP3 was lower in the zinc‐treated group than in the LPS and ATP‐treated group, and 3‐MA application caused a trend compared to that in the zinc‐treated group (Figure 5K). Bafilomycin A1 (BafA1) blocks autophagosome and lysosome fusion to inhibit autophagy and is often used to detect autophagic flux, which was detected by WB in BV2 cells (Figure 5L). The expression of p62 in the zinc‐treated group was lower than that in the Baf A1‐treated group and the 3‐MA‐treated group (Figure 5M). The effect of BafA1 increased the proportion of LC3BII, unlike 3‐MA, which reduced the expression of LC3BII (Figure 5N). All the data showed that after 3‐MA application, the inhibition of the NLRP3 inflammasome was abolished after the zinc‐induced increase in autophagy was blocked. This result indicates that zinc inhibits the NLRP3 inflammasome in LPS‐treated BV2 cells by activating autophagy.

### 3.6 Zinc‐induced ubiquitination facilitates the degradation of the NLRP3 inflammasome

**FIGURE 6:**
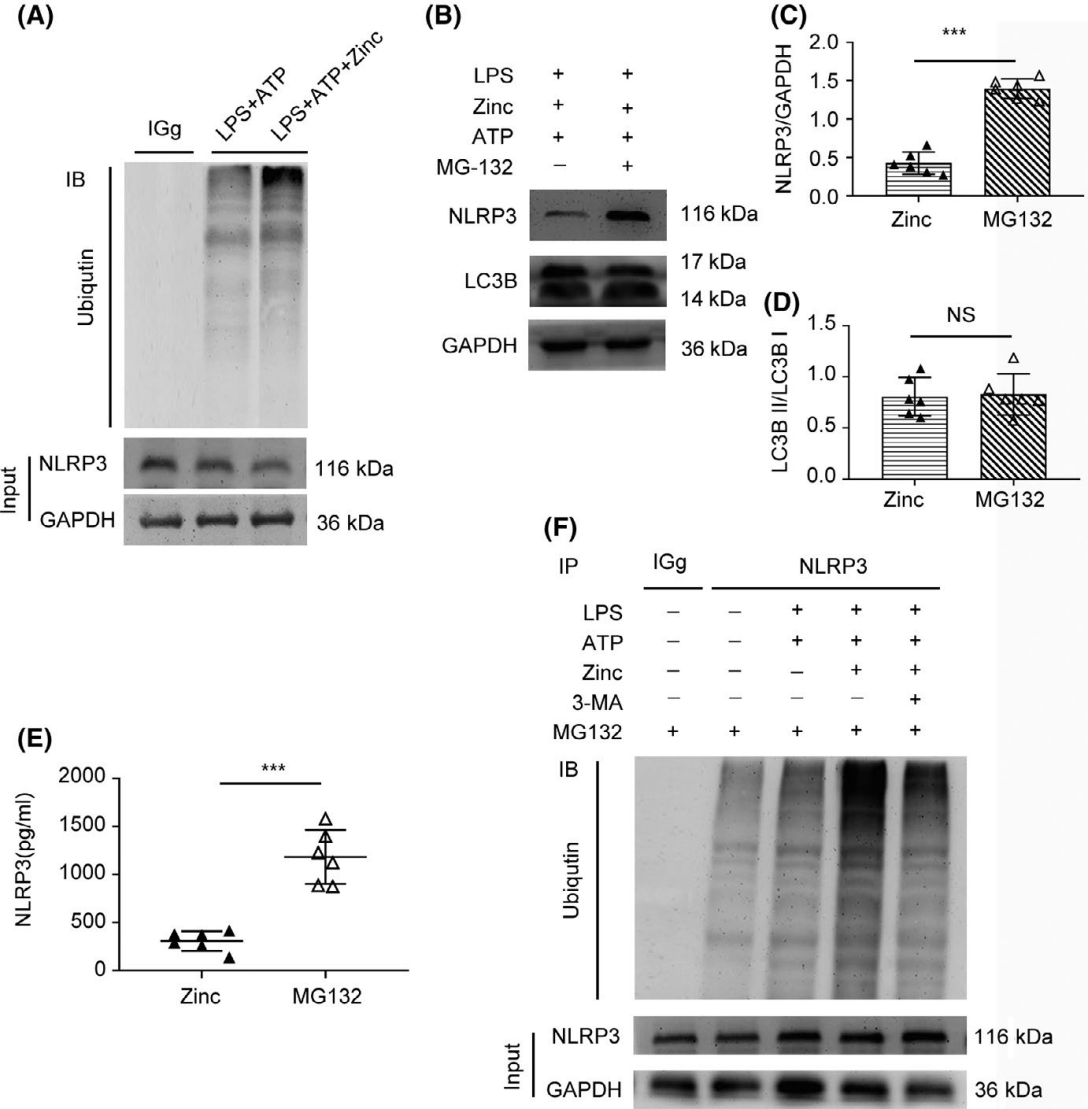
Zinc‐induced ubiquitination facilitates the degradation of the NLRP3 inflammasome (A) Immunoblot analysis of lysates of BV2 exposed to LPS (1 µg/mL) and ATP (5 mmol/L), zinc (100 µmol/L), which was used to detect the interaction between ubiquitin and NLRP3 protein. (B‐D) WB analysis was applied to detect the level of NLRP3 and LC3B after treatment of MG‐132 in BV2 (n = 6, all the data are expressed as means ± SD, Mann‐Whitney U test was applied). (E) NLRP3 was detected by ELISA between the LPS + ATP + zinc group and LPS + ATP + zinc + MG‐132 group (n = 6, all the data are expressed as means ± SD, Mann‐Whitney U test was applied). (F) Co‐IP was used to indicate the relationship of NLRP3 and ubiquitination modify (n = 6). * represents comparison between LPS + ATP + zinc group and LPS + ATP + zinc + MG‐132 group; *** means *P* < .001; NS means no statistically significant

To determine how zinc exerts its anti‐inflammatory effects, we used co‐IP to detect changes in the protein expression of ubiquitin during the process of autophagy. We found that zinc treatment increased the protein level of ubiquitin compared with that in the LPS + ATP‐treated group (Figure 6A). MG‐132, which is a ubiquitin inhibitor, was applied to confirm the effect of zinc (Figure 6B) by WB analysis. Compared with BV2 cells not treated with MG‐132, BV2 cells treated with MG‐132 showed higher NLRP3 protein levels, while LC3B levels did not change significantly (Figure 6C,D). Thus, we used co‐IP to demonstrate the complicated relationship (Figure 6E). The results showed that NLRP3 can bind to ubiquitin, which suggests that zinc treatment can recruit ubiquitin to bind to NLRP3 and cause the degradation of NLRP3 via the ubiquitination modification system. We used ELISA analysis to confirm these results. The level of NLRP3 in LPS‐primed BV2 cells treated with MG‐132 was higher than that in the zinc‐treated group (Figure 6F). All these results indicate that the promotion of ubiquitination by zinc facilitates the degradation of the NLRP3 inflammasome.

## 4 DISCUSSION

Zinc is an essential trace element in organisms and regulates metabolic activities under various physiological and pathological conditions^33^; it is a component of many enzymes and is important for tissue growth, renewal, and repair.^34^ In addition, zinc can also work with various genes and receptors on the cell membrane to protect the membrane.^35^ Its mechanism of action has not been clearly elucidated, but existing studies have shown that zinc deficiency can lead to cell dysfunction, which is not conducive to the prognosis of spinal cord injury.^36^ However, in recent years, researchers have found that zinc is a double‐edged sword.^21^ A certain amount of zinc has a protective effect against spinal cord injury, whereas intracellular zinc overload can cause necrosis of nerve cells. Previous research by our team confirmed that 30 mg/kg/day zinc gluconate in 0.1 mL administered ip has a protective effect against spinal cord injury in mice.^26^ The specific mechanism has not been clearly identified.

In this study, we demonstrated the protective effect of zinc gluconate and the specific mechanism by establishing a model of moderate spinal cord contusion injury in mice. Using BMS scores, Nissl staining and IF analysis of neuron cells, we confirmed that mice treated with zinc showed a significantly increase in motor neuron survival in the anterior horn of the spinal cord, reduced tissue contusion in the injured area and significantly improved motor function after spinal cord injury. These findings were in line with our expectations and further confirm that zinc has a neuroprotective effect. A multifaceted approach may be necessary to explore in depth how zinc exerts its neuroprotective effect. To this end, we specifically evaluated the regulatory effect of zinc on the NLRP3 inflammasome in microglia.

As the first line of defense for innate immunity in the nervous system, microglia have different functions at different stages after spinal cord injury.^37^ Previous studies have shown that microglia/macrophages play a crucial role in the activation of inflammation during secondary injury.^26^ NLRP3, which can be expressed by microglia after activation, has long been known as a key protein that induces the expression of inflammatory chemokines.^38^ Our research proved that on the third day after spinal cord injury, the expression of the NLRP3 inflammasome, as a major inflammasome, was significantly increased after injury and that this increase was accompanied by a significant increase in inflammatory marker proteins; these changes are not conducive to recovery from spinal cord injury. Interestingly, this phenomenon was suppressed after zinc treatment. The application of zinc reduced the expression of NLRP3, ASC, Caspase‐1, and IL‐1β and increased the levels of autophagy‐related proteins, which suggested that the application of zinc inhibits the expression of NLRP3 and exerted an antineuroinflammatory effect. This effect can be regulated by autophagy levels. To further clarify this complex relationship, we applied 3‐MA and BafA1 for in vitro interference experiments. The co‐administration of LPS and ATP successfully induced the expression of NLRP3 in BV2 cells, and we verified the protective effect of zinc gluconate on the inflammatory phenotype of BV2 cells using the MTT assay. The results of these experiments suggested that the zinc‐induced activation of autophagy can be blocked by autophagy inhibitors and that after this inhibition, the inhibitory effect of zinc on NLRP3 is abolished, confirming that zinc‐induced autophagy can negatively regulate the NLRP3 inflammasome. Previous research has shown that autophagy and the UPS can cooperate with each other to completely eliminate the abnormal accumulation of proteins.^39^ We found through co‐IP that the application of zinc promoted the binding of ubiquitin to NLRP3, which seems to suggest that zinc can also activate the UPS to have an anti‐inflammatory effect. To verify this conclusion, we applied the ubiquitin protein inhibitor MG‐132. The results showed that, under zinc treatment, when the ubiquitination was blocked, the levels of NLRP3, but not the levels of autophagy‐related proteins, were increased. Further experiments showed that after 3‐MA was used to inhibit autophagy, the degree to which ubiquitin bound NLRP3 decreased significantly. Unfortunately, because co‐IP results did not provide any evidence for the binding of autophagy‐related proteins to ubiquitin, our research data cannot explain how the formation of autophagosomes affects ubiquitination. Taken together, these findings suggest that zinc regulates the NLRP3 inflammasome through autophagy and ubiquitination to provide neuroprotection in spinal cord contusion models.

Although we believe that the data obtained in this experiment are very convincing and provide valuable theoretical guidelines for the clinical treatment of SCI, there are two limitations to this study. First, the research study was not carried out on transgenic mice with zinc transporter knockout due to technological limitations. The use of these mice could provide more reliable and convincing experimental conclusions and could further determine the proper dose of zinc and how exert its effects on autophagy activation and inflammation. Second, in this study, we did not conduct experimental research in the clinic. Therefore, although we have demonstrated that zinc has anti‐inflammatory effects in spinal cord injury recovery in mice, whether it has the same effect in the human body remains unknown.

## 5 CONCLUSION

We have demonstrated that zinc can regulate NLRP3 inflammasome by activating autophagy and ubiquitination modification. Our findings indicate that zinc's treatment can protect nerve cells after spinal cord injury and promote motor function recovery providing a new promising treatment strategy for the treatment of spinal cord injury in clinic.

## CONFLICT OF INTEREST

The authors declare that there are no conflicts of interest.

## AUTHOR CONTRIBUTIONS

J.L and H.T completed the conception and design of the whole experiment. J.L, S.L, X.Z, Y.L, and D.L were involved in behavioral scoring and sample preparation. J.L, C.X, and S.L were involved with cell cultures. J.L and H.T finished statistical analysis and manuscript preparation. Prof X.M finished the final review and submitted the manuscript. All authors provided important intellectual content to the manuscript and approved to its publication.

## Supporting information

Fig S1

Fig S2

Supplementary Material

## Data Availability

The data that support the findings of this study are available from the corresponding author upon reasonable request.
