# Supplementary figures and images for: Zinc provides neuroprotection by regulating NLRP3 inflammasome through autophagy and ubiquitination in a spinal contusion injury model
Source: CNS Neurosci Ther. 2020 Oct 9;27(4):413–25. doi: 10.1111/cns.13460 (PMC7941232; doi:10.1111/cns.13460)

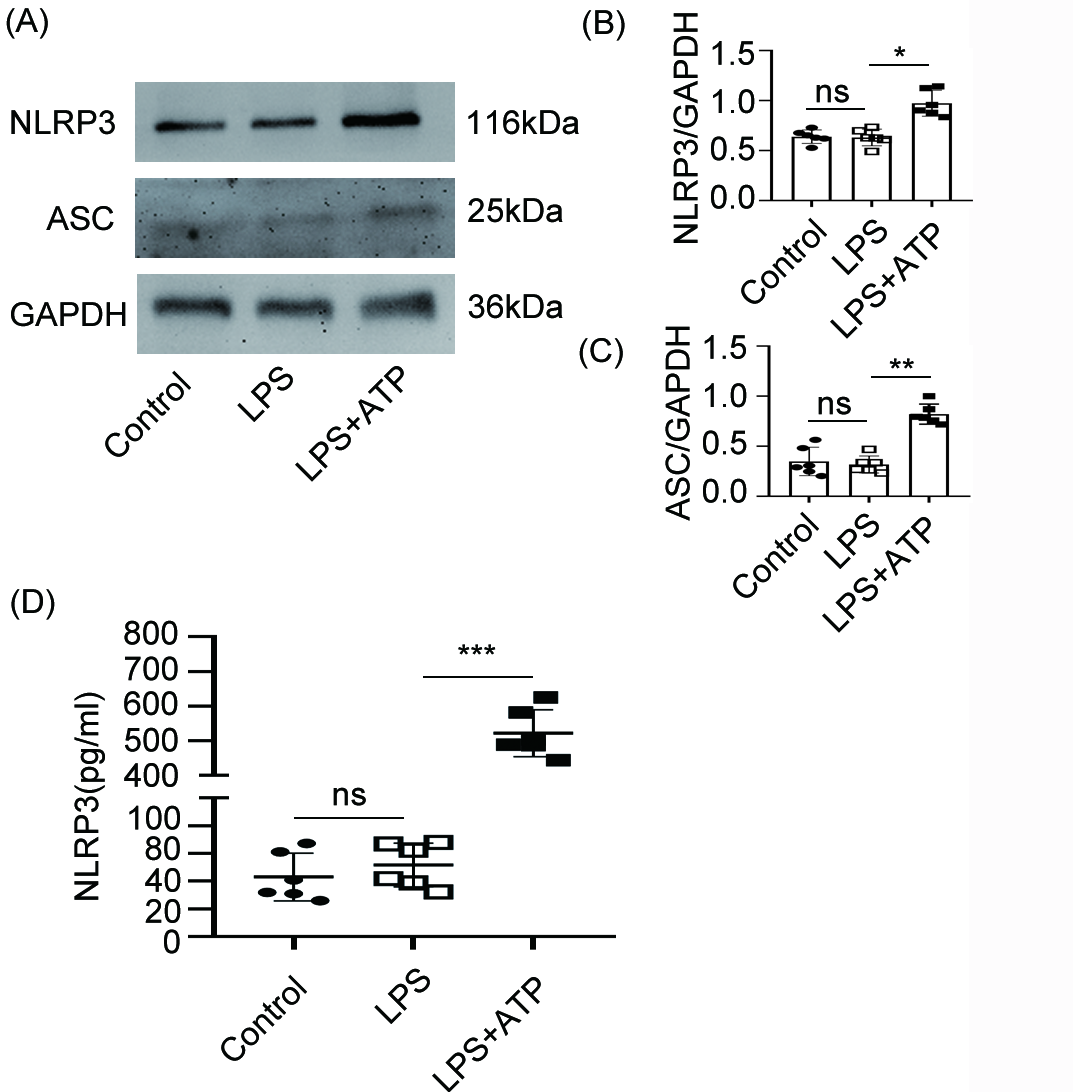

Supplement: Supplementary file 1 — Fig S1 [file CNS-27-413-s003.tif]

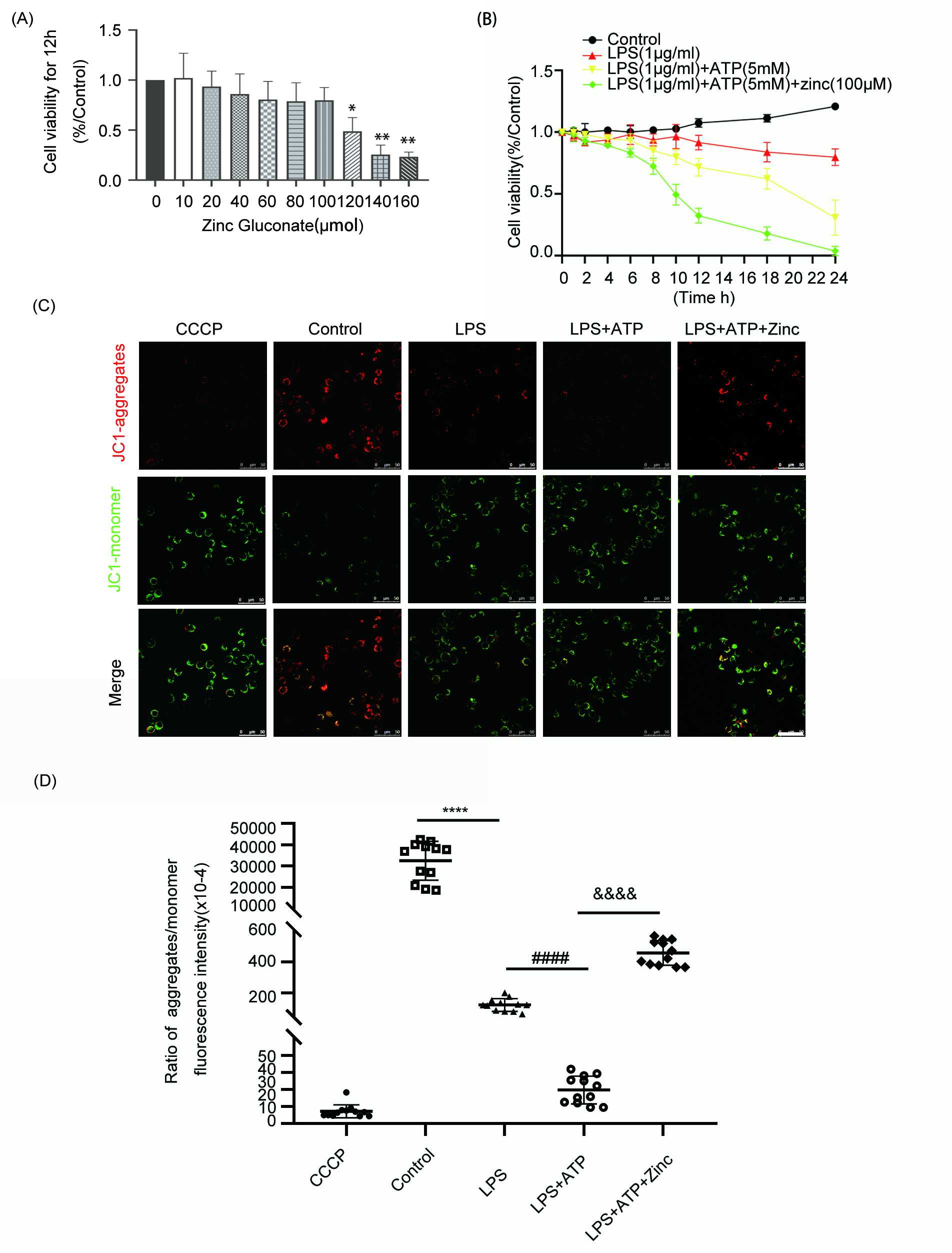

Supplement: Supplementary file 2 — Fig S2 [file CNS-27-413-s002.tif]
