# Supplementary material for: Zinc provides neuroprotection by regulating NLRP3 inflammasome through autophagy and ubiquitination in a spinal contusion injury model
Source: CNS Neurosci Ther. 2020 Oct 9;27(4):413–25. doi: 10.1111/cns.13460 (PMC7941232; doi:10.1111/cns.13460)
Supplement: Supplementary file 3 — Supplementary Material [file CNS-27-413-s001.docx]

**Supplemental method**

**1. MTT assay**

Cells were cultured and treated in 96-well plates as described above. Cells in logarithmic growth were digested with 0.25% trypsin, and the reaction was stopped by the addition of 1:1 DMEM:10% FBS. The cells were centrifuged at 1000 rpm for 5 minutes at 4°C, the supernatant was aspirated, and medium was added to the cell pellet. The cells were mixed well to make a cell suspension. The cells were counted and plated in 96-well plates at a density of 5-10×10^4^ cells/ml. After the cells were cultured and treated as described above, 10 μL MTT solution (5 mg/ml) was added to each well, and the cells were cultured in a cell incubator for 4-6 h. The medium was carefully aspirated, and 150 μL DMSO was added to each well, and the cells were incubated with shaking at low speed for 10 min in the dark to fully dissolve the crystals. Finally, a microplate reader was used to detect the absorbance of each well at 570 nm.

1. **JC-1 staining**

We followed the procedure of JC-1 Staining Kit (Solarbio,Cat#M8650,CN) to finish the JC-1 staining. Briefly described as follows: Plant 5*10^5^ cells in a 12-well plate and treat the cells according to the above method.Then, add 250ul JC-1 fluorescent probe and incubate at 37℃ for 1 hour. Next, remove the supernatant and wash twice with staining buffer for 2 minutes per time. Finally, add 500ul culture medium to each well and observed, imaged with a high-resolution confocal microscope.
